# Dose-Dependent Effects of Astaxanthin on Exercise-Induced Muscle Damage in Exercising Males

**DOI:** 10.5114/jhk/210201

**Published:** 2026-04-02

**Authors:** Muhammet Yılmaz, Halit Harmancı, Filiz Özyiğit, Muhammed M. Atakan

**Affiliations:** 1 Department of Physical Education and Sports Teaching, Faculty of Sport Sciences, Sabahattin Zaim University, Istanbul, Turkiye.; 2 Department of Coaching Education, Faculty of Sport Sciences, Dumlupinar University, Kutahya, Turkiye.; 3 Department of Medical Pharmacology, School of Medicine, Onyedi Eylul University, Bandirma, Balikesir, Turkiye.; 4 Division of Exercise Nutrition and Metabolism, Faculty of Sport Sciences, Hacettepe University, Ankara, Turkiye.*

**Keywords:** supplements, antioxidant, resistance exercise, carotenoids

## Abstract

Astaxanthin (AX) is a potent antioxidant and an anti-inflammatory carotenoid. Research examining whether AX could counteract exercise-induced muscle damage and improve exercise capacity has reported inconsistent results. The aim of this study was to test the efficacy of high-dose versus low-dose AX supplementation for four weeks on muscle damage markers, total antioxidant status, and a subjective marker of muscle pain following exhaustive exercise. A total of 24 active males were randomly assigned to one of the three groups: an AX12 group (12 mg·day^−1^; n = 8), an AX36 group (36 mg·day^−1^; n = 9) or a placebo group (PLC, n = 7). After four weeks of supplementation, blood samples were collected at rest, and at 2, 24, 48, and 72 h following eccentric arm exercise performed at 85% of the predetermined one-repetition maximum to assess muscle damage markers and total antioxidant status, and muscle pain levels were evaluated using a Numerical Visual Pain Scale_0–10_. Creatine kinase activity was significantly lower in AX groups compared to the PLC group at 24, 48, and 72 h post-exercise (p < 0.05), with no difference between both AX groups (p > 0.05). At 24, 48, and 72 h post-exercise, lactate dehydrogenase activity in the PLC group was higher than in AX12 and AX36 groups, averaging 2.2 and 2.8 times higher, respectively; however, these differences were not statistically significant (p > 0.05). A significant time effect of the muscle pain score was noted at 2, 24, 48, and 72 h post-exercise (p < 0.001), with no significant differences among the supplementation protocols (p > 0.05). In conclusion, four-week AX supplementation at a dose of 12 or 36 mg·day^−1^ similarly reduces plasma creatine kinase activity following exhaustive exercise, yet its impact on muscle pain and antioxidant status remains limited.

## Introduction

Excessive or intense exercise performed beyond physical limits can cause various musculoskeletal damage (Howatson and van Someren, 2008). Such damage typically occurs during eccentric contractions, where the muscle lengthens, while resisting an external force that exceeds its force-generating capacity (Howatson and van Someren, 2008). In general, muscle damage caused by these types of muscle contractions occurs following high-intensity exercises (Markus et al., 2021; Podgórski et al., 2021; Ponce-Gonzalez et al., 2021). However, the mechanisms causing exercise-induced muscle damage are indeed complex. Nonetheless, three key factors that significantly contribute to this damage are the generation of free radicals, hypoxia-induced ATP consumption, and the disruption of intracellular calcium homeostasis (Bloomer, 2007; McArdle et al., 2002; Paoli et al., 2024; Viña et al., 2000).

During strenuous exercise, endogenous antioxidant compounds such as superoxide dismutase, catalase, and glutathione peroxidase work together to prevent the harmful effects of reactive oxygen species (ROS) on the organism (He et al., 2016). However, if the exercise becomes very intense for the organism to handle, excessive production of reactive oxygen and nitrogen species can overwhelm the antioxidant defense system, leading to oxidative stress (Viña et al., 2000). Indeed, an overproduction of ROS, combined with the failure of antioxidant systems, can result in a loss of redox homeostasis and oxidative damage to cellular lipids, proteins, and DNA (McArdle et al., 2002; Viña et al., 2000).

There has been increasing interest in dietary antioxidants used as exogenous supplements to reduce muscle damage or oxidative stress after high-intensity exercise by inhibiting the formation of reactive oxygen and nitrogen species (Bongiovanni et al., 2020; Kuru et al., 2024). Antioxidant supplements may not directly modulate this inflammatory process; however, they could help alleviate tissue damage, acute strength loss, oxidative stress, and delayed onset muscle soreness (DOMS) associated with eccentric exercise (Bongiovanni et al., 2020). Among the strategies to prevent ROS production and muscle damage, vitamin C, vitamin E, and carotenoids are widely used antioxidant supplements (Bloomer, 2007; Bongiovanni et al., 2020; Paoli et al., 2024). To date, more than 800 carotenoids have been documented (Rodriguez-Amaya, 2015) and among these, astaxanthin (AX) has received considerable interest in recent years in the field of antioxidant supplements (Cao et al., 2023; Higuera-Ciapara et al., 2006). AX is a naturally occurring, lipid-soluble carotenoid predominantly sourced from microalgae and marine species and has been considered a dietary supplement due to its powerful antioxidant properties (Brown et al., 2017; Miki, 1991). Current research suggests that AX may modulate inflammation by influencing key transcription factors such as NF-κB and Nrf2 (Chang and Xiong, 2020), reduce markers of oxidative stress (Fassett et al., 2008), improve total antioxidant capacity (Choi et al., 2011; Hasani et al., 2024), suppress lactic acid accumulation during high-intensity exercise (Wu et al., 2019), and improve fat metabolism (Aoi et al., 2008). These properties make AX a promising exercise performance-enhancing agent, improving high-intensity exercise performance and facilitating post-exercise recovery.

The post-exercise recovery process is crucial for optimizing the effectiveness and quality of subsequent training sessions for athletic performance and recreational health purposes. AX, due to its potent antioxidant properties, may help mitigate exercise-induced damage and indirectly accelerate recovery by inhibiting pro-oxidant and pro-inflammatory intermediates (Brotosudarmo et al., 2020; Brown et al., 2018). However, findings from human studies remain inconsistent (Choi et al., 2011; Djordjevic et al., 2012; Fleischmann et al., 2019; Klinkenberg et al., 2013; Park et al., 2010; Res et al., 2013; Waldman et al., 2023; Wu et al., 2019), while animal studies provide more consistent evidence supporting AX as a potential ergogenic aid (Aoi et al., 2008; Brown et al., 2017; Cao et al., 2023; Wang et al., 2023). This discrepancy highlights the need for more rigorous research to draw firmer conclusions about AX’s effectiveness in humans.

Hitherto, research on the metabolic effects of AX supplementation on exercise-induced muscle soreness in humans is limited and has yielded inconsistent results. For instance, supplementation for three to four weeks at doses of 4, 12, or 20 mg·day^−1^ did not significantly affect markers of muscle damage, inflammation or DOMS in trained male participants undergoing muscle-damaging exercise protocols (Bloomer et al., 2005; Klinkenberg et al., 2013; Waldman et al., 2023). In contrast, Wu et al. (2019) reported a reduction in free radical production following high-intensity acute exercise after four weeks of AX supplementation at a dose of 12 mg·day^−1^ in trained males. Similarly, Djordjevic and colleagues (2012) found reduced plasma aspartate aminotransferase and creatine kinase (CK) activity after twelve weeks of 4 mg·day^−1^ AX supplementation in elite soccer players. These inconsistent findings may be explained by variations in participants’ training status, the type, dosage, and duration of AX supplementation, and differences in exercise modality (e.g., endurance, resistance), intensity, and duration. These factors could influence the degree of oxidative stress and muscle damage, ultimately affecting the potential efficacy of AX supplementation. Therefore, in particular, further research is warranted to determine whether increasing the dosage of AX supplementation (e.g., 36 mg·day^−1^) can enhance its efficacy over shorter duration (e.g., four weeks). Indeed, a recent systematic review with meta-analysis showed that compared to lower dosage of supplementation, AX supplementation provided greater benefits when the dose was ≥ 20 mg·day^−1^ (Liu et al., 2024). Therefore, the aim of this study was to evaluate the efficacy of high-dose AX supplementation (36 mg·day^−1^) compared to a low dose (12 mg·day^−1^) over short duration (four weeks) on markers of muscle damage, total antioxidant status (TAS), and a subjective marker of muscle pain following a single session of eccentric arm exercise in active males.

## Methods

### Participants

**Figure 1:**
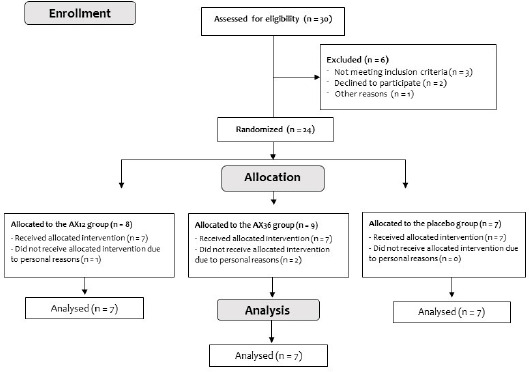
Consolidated standards of reporting trials (CONSORT) flow diagram of study participation from enrollment to analysis. AX: astaxanthin

Twenty-four young, healthy males with prior resistance training experience (age: 20.91 ± 1.5 years, body mass: 72.4 ± 9.8 kg, body height: 174.0 ± 5.6 cm), who participated in recreational endurance-type exercise (e.g., cycling, running, soccer) 2–3 times per week, volunteered for this randomized, placebo-controlled study. The inclusion criteria comprised males aged 20 to 30 years who engaged in recreational physical activity, were non-smokers, and did not participate in any structured training regimen (e.g., endurance or resistance training). Exclusion criteria included allergies related to supplement use, regular medication use, diagnosed hypothyroidism or hyperthyroidism, and the presence of orthopedic, metabolic, or locomotor system disorders. This group of participants was intentionally recruited because they could train at the volume and intensity necessary to induce DOMS as well as represent the target population for dietary supplements aimed at reducing soreness or enhancing recovery. Before study enrollment, each participant attended a face-to-face meeting where the study procedures were thoroughly explained. During this meeting, participants were also informed that they would be required to abstain from any structured exercise programs throughout the study, including resistance, endurance, or concurrent exercise, from one week before supplementation until the final blood sample collection (i.e., 72 h after completing the exercise protocol following post-supplementation). Only participants who explicitly confirmed their willingness to comply with this condition were included in the study. Participants were randomly assigned to one of the three groups for four weeks: 12 mg·day^−1^ AX (AX12; n = 8), 36 mg·day^−1^ AX (AX36; n = 9), and placebo (PLC; n = 7) groups. Participant flow through the study is presented in the consolidated standards of reporting trials (CONSORT) diagram (Figure 1). All participants were asked to complete a questionnaire concerning their medical history, dietary habits, and supplement usage. Participants signed an institutionally approved informed written consent document after receiving explanations of all procedures, risks, and benefits. The recruitment period for this study commenced on the 20^th^ of November 2023 and concluded on the 10^th^ of March 2024. The study conformed to the Declaration of Helsinki and was approved by the Istanbul Medipol University GETAT Clinical Research Ethics Committee, Istanbul, Turkey (approval number: E-95961207-604.01.01-2244; approval date: 30 March 2023). This study was registered at ClinicalTrials.gov (NCT06511960).

### Study Design

The present study followed a placebo-controlled, double-blind (participants and co-researcher administering the supplementation), and three-group parallel design to examine the effects of four-week AX supplementation on muscle pain, muscle damage markers, and TAS. Participants reported to the laboratory on two separate occasions. During the initial visit, body mass (Tanita MC 780 ST Black), body height (Tarti Telescopic Height Meter), and one-repetition maximum (1-RM) strength of the arm muscles were measured. Resting venous blood samples were then collected from the median antecubital vein by a healthcare professional prior to the four-week supplementation period. Following these measurements, participants were assigned to one of the groups through a strict randomization process that involved selecting from boxes numbered up to 24, all identical in size, color, and the pattern. Participants were asked to choose one of the random numbers accompanied by an independent person other than the researchers. This process was recorded by the supervisor in paper-based surveys and digitally. Subsequently, all participants were instructed to consume their corresponding supplements, i.e., 12 mg·day^−1^ AX, 36 mg·day^−1^AX, or 36 mg·day^−1^ PLC, for four weeks.

After the four-week supplementation period, participants reported to the laboratory for post-supplementation measurements that included the collection of blood samples at rest and at 2, 24, 48, and 72 h following eccentric arm exercise performed at 85% of the predetermined 1-RM. Before obtaining blood samples, participants were also asked to assess their current pain levels using the Numerical Visual Pain Scale (NRS, ranging between 0–10 and 0–100) to determine any correspondence with muscle damage markers in the blood (Haefeli and Elfering, 2006). All participants were instructed to abstain from taking any medications for a minimum of seven days prior to the study and to avoid consuming vitamins, foods, or supplements containing antioxidants, as well as analgesics, aspirin, or any other anti-inflammatory drugs throughout the study. A list of AX-rich foods, beverages and vitamins was provided to each participant before testing and all participants were instructed to avoid these items during the four-week supplementation period, and to maintain their regular eating habits and refrain from strenuous exercise to minimize the risk of muscle damage. A WhatsApp group and a Google Forms survey were created to monitor participants’ supplement use and assess compliance. Supplement intake was tracked daily during the supplementation period, and the submitted data were reviewed each day by at least one researcher using an Excel file shared via OneDrive.

### Supplementation Protocol

In a double-blind fashion, participants ingested either 12 mg·day^−1^ (AX12) or 36 mg·day^−1^ (AX36) of AX at 8-h intervals to ascertain an intake of 12 or 36 mg AX per day for four weeks. We selected 12 mg·day^−1^ as the minimum effective dose based on earlier research (Waldman et al., 2023; Wu et al., 2019), and 36 mg·day^−1^ to approximate the upper range of doses used in previous human studies (e.g., up to 40 mg·day^−1^) (Kupcinskas et al., 2008; Mercke Odeberg et al., 2003), albeit with different exercise protocols. Unlike previous studies that administered the daily dose as a single serving, we divided the total daily amount of AX into three doses, served every 8 h, to potentially enhance bioavailability and optimize antioxidant capacity. Specifically, the supplementation boxes were prepared with either 4 mg or 12 mg of AX to be taken every 8 h, resulting in total daily doses of 12 mg·day^−1^ or 36 mg·day^−1^ AX, respectively. For the PLC group, the boxes were designed to match the high AX dose schedule (i.e., 36 mg·day^−1^), with capsules containing 12 mg of the placebo to be taken every 8 h, totaling 36 mg·day^−1^ of the PLC. The AX capsules were provided by OCEAN (Orzaks Pharmaceuticals and Chemical Industry Co. Ltd., Istanbul, Turkiye). Each 4 mg AX capsule produced from Haematococcus Pluvialis contained sunflower oil, gelatin, AX, deionised water and glycerol. The PLC capsules were identical in appearance and dimensions to the AX supplement. All supplements were dispensed to participants by an independent individual immediately after the collection of their first blood sample during their initial visit. Participants were instructed to take AX supplements with a full stomach. Additionally, to enhance compliance, a researcher contacted participants every eight hours via phone or WhatsApp, scheduling messages to align with typical waking hours while allowing flexibility in cases where participants were asleep during a scheduled reminder. No adverse effects related to supplementation were reported by participants. Supplementation compliance was assessed by the number of pills that were remaining in the pill bottle after the four-week supplementation period, with a compliance ratio > 90%.

### Measurement of One-Repetition Maximum (1-RM) Arm Strength

At least 72 h before the supplementation protocol, the 1-RM for non-dominant arm strength was assessed using the multiple-repetition test, following the procedure outlined by Brown and Weir (2001), with minor modification. Briefly, following a demonstration of the lifting technique, participants performed a warm-up set of 8 to 10 repetitions at approximately 50% of the estimated 1-RM. Afterward, they completed a set of 5 repetitions of the biceps curl at 75% of the estimated 1-RM. After a two-minute recovery period, a second set of 3 repetitions was performed at approximately 90% of the 1-RM. Following a four-minute rest interval, incremental 2.5-kg increases were applied to each subsequent 1-RM attempt until failure. The maximum weight successfully lifted was recorded as the 1-RM for non-dominant arm strength (Brown and Weir, 2001). The rationale for selecting the non-dominant arm was to introduce relatively unfamiliar exercise stress and to augment the rate of muscle damage by increasing mechanical stress during the eccentric arm exercise.

### Application of Eccentric Arm Exercise following Supplementation

After four weeks of supplementation, all participants performed an eccentric arm exercise that consisted of 10 sets × 10 repetitions at 85% of 1-RM, with a 3-min passive rest interval between each set, following a 10-repetition warm-up with 50% of 1-RM. During the exercise, participants freely positioned their dominant arm either at their side or on their knee, while the elbow of the non-dominant arm was rested on the knee joint of the same leg. Resistance was provided by an assistant when the arm reached full flexion. Participants were then instructed to move the weight, positioned at full flexion, to the point of full elbow extension within 3–5 s to induce maximal mechanical stress on muscle fibers.

### Assessment of Muscle Pain

The pain sensation in the participants' elbow extensor and elbow flexor muscles was assessed using the NRS. The NRS is one of the most widely used scales due to its simplicity and effectiveness in assessing pain intensity across various medical settings and research studies (Haefeli and Elfering, 2006). This scale rates individuals’ pain on a scale from 0 to 10, with 0 representing no pain and 10 representing the worst pain imaginable (1–3: mild pain, 4–6: moderate pain, 7–10: severe pain). In this study, we evaluated the participants' pain at 2, 24, 48, and 72 h after eccentric arm exercise, following completion of the supplementation protocol. For this, participants were instructed to move their arms, which were used in the 1-RM eccentric arm exercise, from a fully bent position to a fully straight position. During this movement, participants were asked to rate the intensity of pain they experienced.

### Blood Sampling and Analysis

Resting venous blood samples were collected from the antecubital vein before and after the four-week AX supplementation period. Additionally, blood samples were obtained before, and at 2, 24, 48, and 72 h after the eccentric arm exercise (10 sets of 10 repetitions at 85% of 1-RM) performed at the end of the supplementation program. Oxidative stress markers (TAS, malondialdehyde [MDA], and uric acid) and muscle contraction markers (CK and lactate dehydrogenase activity [LDH]) were analyzed from the collected blood samples. In addition, as an exclusion criterion, C-reactive protein (CRP) analysis was performed immediately before exercise and 24 h after exercise as evidence of the presence of any infectious disease in the body. The average concentrations of CRP were 0.8 mg·L^−1^ before exercise and 1.90 mg·L^−1^ 24 h after exercise. The collected blood samples were left at room temperature for approximately 30 min before being centrifuged at +4°C and 3000 rpm for 20 min using a high-speed refrigerated centrifuge (Himac CR22N, Hitachi Koki Co., Ltd., Tokyo, Japan). Following centrifugation, the transparent liquid remaining at the top of the tube was transferred to 2-ml Eppendorf tubes. These tubes were then stored at −20°C until blood analysis.

### Measurement of Malondialdehyde (MDA)

MDA is a natural product of lipid peroxidation. Lipid peroxidation is a well-known mechanism of animal and plant cell damage indicating the level of oxidative stress or damage in cells and tissues (Zadeh-Ardabili et al., 2017). The MDA concentrations were assessed using thiobarbituric acid reactive substances assay kits (Cayman TBARS, Cayman Chemical, Ann Arbor, Michigan, USA, item no. 10009055) based on the trichloroacetic acid method in accordance with the manufacturer's instructions. The absorbance measurements were taken using the ChemWell 2910 ELISA reader device (Awareness, Technology, Inc. Martin Hwy. Palm City, USA). The results were presented as µM (Porter, 1984).

### Measurement of CRP, CK, LDH and Uric Acid

Serum CRP, CK, LDH and uric acid were analyzed on a fully automatic analyzer (Roche Cobas Integra 400 Plus, Roche Diagnostics GmbH, Mannheim, Germany) using ROCHE kits (Mannheim, Germany). Data were calculated using linear regression and measurements were taken at 550 nm.

### Measurement of Total Antioxidant Status (TAS)

Serum TAS was determined with the Rel Assay Diagnostics kit (Mega Tip, Gaziantep, Turkey) using the method developed by Erel (2004). This method mediates the production of a hydroxyl radical. The hydroxyl radical is the most powerful one among biological radicals. In the test, the Fe ion solution present in reagent 1 is mixed with hydrogen peroxide present in reagent 2. Radicals formed sequentially, such as the brown dianisidinyl radical cation produced by the hydroxyl radical, are also among the strong biological radicals. Using this method, the antioxidative effect of the sample against the strong free radical reactions initiated by the hydroxyl radical produced was measured (Erel, 2004). The test had excellent sensitivity values of > 97%. Results were expressed as millimoles of Trolox equivalents per liter (mmol Trolox Equiv.·L^−1^).

### Statistical Analysis

A two-way analysis of variance (ANOVA) was conducted to examine the effects of four weeks of supplementation with AX12, AX36, and the PLC on muscle pain scores, muscle damage markers, and TAS at different time points (i.e., 2, 24, 48, and 72 h) following a single session of exhausting acute resistance exercise. Following ANOVA, post-hoc comparisons were performed using the Tukey's post-hoc test to determine specific group differences. All data are presented as mean ± standard deviation (SD). Considering that our small sample size did not permit definitive analysis, this statistical analysis of the data was exploratory only. A power analysis was not performed due to limited data on AX; therefore, the sample size was determined based on previous studies investigating similar supplements and related blood markers (Bloomer et al., 2007; Earnest et al., 2011; Wu et al., 2019). Statistical analyses were computed using Statistical Package for the Social Sciences (SPSS) for Windows, Version 21.0 (IBM Corp., Armonk, NY, USA), and the level of significance was set at *p* < 0.05.

## Results

The characteristics of participants are shown in Table 1. Three participants left the study prematurely; thus, twenty-one participants, including seven from each group, completed the study in its entirety and were included in the analysis. Exclusion from the final analysis was due to difficulties in adhering to the meal schedule, inability to follow the supplementation protocol, and failure to attend required blood sampling sessions due to work-related constraints. At the beginning of the study, there were no statistically significant differences among the groups in terms of age, body mass, height, body mass index, 1-RM, or use of supplement before the commencement of the study (*p* > 0.05; Table 1).

**Table 1:** Participants’ characteristics.

Variables	AX12 (n = 7)		AX36 (n = 7)		PLC (n = 7)		*p* (for all)
	Mean ± SD	Range	Mean ± SD	Range	Mean ± SD	Range	
Age, years	21.0 ± 1.6	18.0–23.0	20.9 ± 2.2	19.0–25.0	20.8 ± 1.6	18.0–25.0	ns
Body mass, kg	68.8 ± 11.5	53.5–91.0	73.0 ± 8.0	62.2–89.0	71.8 ± 9.6	53.5–91.0	ns
Body height, cm	172.1 ± 4.5	166.0–180.0	174.5 ± 6.4	168.0–187.0	174.1 ± 5.7	166.0–187.0	ns
BMI, kg·m^−2^	23.1 ± 3.1	19.4–28.0	24.0 ± 2.5	20.3–28.0	23.9 ± 1.8	19.1–21.6	ns
1-RM, kg	15.0 ± 3.0	10.0–18.0	15.8 ± 2.8	12.0–21.0	15.9 ± 3.1	10.0–22.0	ns
1-RM 85%	12.5 ± 2.3	8.5–15.3	13.6 ± 2.3	10.2–17.8	12.6 ± 3.7	8.5–18.7	ns
Use of supplement	1.5 ± 1.1	1.0–4.0	1.7 ± 0.7	1.0–3.0	1.67 ± 0.9	1.0–4.0	ns

AX: astaxanthin, BMI: body mass index, Ns: non-significant, PLC: placebo, SD: standard deviation, 1-RM: one-repetition maximum

### Changes in Muscle Pain Scores

**Figure 2:**
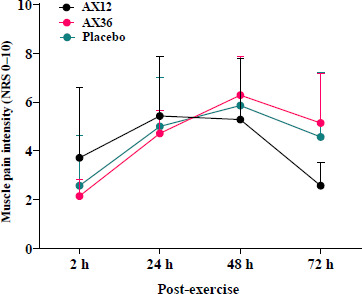
Mean (± SD) NRS scores of the pain intensity ratings for groups. NRS: numerical rating scale, AX: astaxanthin

The results of the two-way ANOVA revealed a significant time effect of the muscle pain score measured via the NRS at 2, 24, 48, and 72 h following eccentric arm exercise performed at 85% of the predetermined 1-RM (*p* < 0.001). No significant differences were noted among the supplementation protocols (*p* > 0.05; Figure 2), suggesting the different doses of AX supplementation did not result in significant changes in levels of muscle pain at these time points.

### Changes in Blood Markers

**Figure 3:**
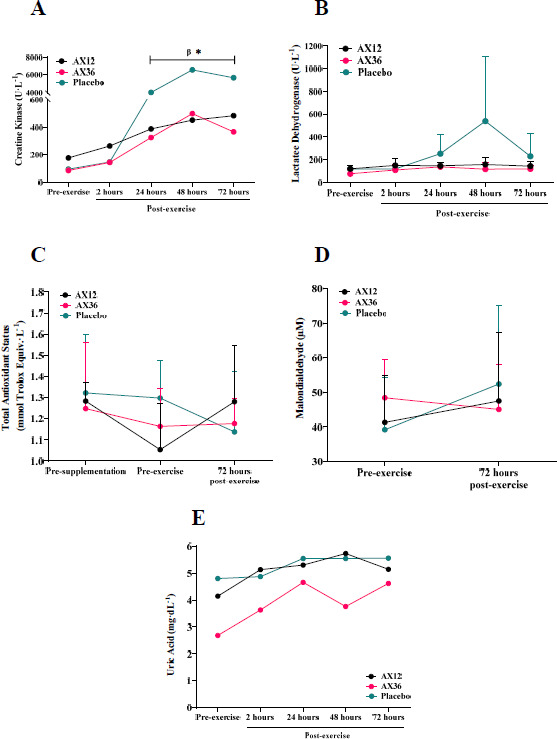
Changes in creatine kinase (A), lactate dehydrogenase (B), total antioxidant status (C), malondialdehyde (D), and uric acid (E). AX: astaxanthin. *: Significant difference (p < 0.05) between AX12 and PLC groups at 24, 48, and 72 h post exercise; β: Significant difference (p < 0.05) between AX36 and PLC groups at 24, 48, and 72 h post exercise

CK activity was significantly lower in the AX12 and AX36 groups compared to the PLC group at 24, 48, and 72 h post-eccentric arm exercise (*p* < 0.05), with no notable difference between the two AX groups (*p* > 0.05; Figure 3A). No significant differences in CK activity among the groups were observed at baseline or 2 h post-exercise (*p* > 0.05; Figure 3A). At 24 h post-exercise, LDH activity was 1.7 and 1.8 times higher in the PLC group compared to the AX12 and AX36 groups, respectively. This difference persisted at 48 h post-exercise (3.4 and 4.6 times higher) and 72 h post-exercise (1.6 and 1.9 times higher) (Figure 3B); yet these differences were not statistically significant (*p* > 0.05; Figure 3B). There was no statistically significant difference in TAS, MDA, or uric acid concentrations at the measured time points after AX or PLC supplementation (*p* > 0.05; Figure 3C–E). The overall changes in blood markers are presented in Table 2.

**Table 2:** Overall changes in blood markers in astaxanthin and placebo groups (mean ± standard deviation).

Variables	AX12 (n = 7)	AX36 (n = 7)	Placebo (n = 7)	*p* ^a^	*p* ^b^	*p* ^c^
Serum CK (U·L^−1^)						
Pre-exercise	178.6 ± 136.6	86.8 ± 80.9	96.3 ± 43.5	0.263	0.368	0.99
2 h post-exercise	264.8 ± 158.1	145.4 ± 13.8	148.6 ± 68.0	0.298	0.314	0.99
24 h post-exercise	388.6 ± 314.8	236.0 ± 216.7	4025.6 ± 4386.2	0.99	0.046*	0.042*
48 h post-exercise	543.0 ± 623.6	500.3 ± 637.2	6567.6 ± 6419.8	0.99	0.020*	0.021*
72 h post-exercise	485.1 ± 472.8	367.8 ± 363.3	2269.4 ± 3872.8	0.99	0.017*	0.019*
Serum LDH (U·L^−1^)						
Pre-exercise	120.4 ± 30.3	76.6 ± 35.5	118.0 ± 18.6	0.033*	0.99	0.047*
2 h post-exercise	149.6 ± 58.3	110.0 ± 45.8	118.8 ± 42.5	0.478	0.779	0.99
24 h post-exercise	146.6 ± 32.5	137.4 ± 18.3	253.0 ± 166.0	0.99	0.173	0.123
48 h post-exercise	158.1 ± 60.8	116.7 ± 54.7	538.4 ± 568.8	0.99	0.138	0.086
72 h post-exercise	144.0 ± 41.8	118.8 ± 30.0	230.0 ± 202.2	0.99	0.633	0.374
Serum uric acid (mg·dL^−1^)						
Pre-exercise	4.1 ± 1.5	2.7 ± 1.3	4.8 ± 0.7	0.105	0.953	0.012*
2 h post-exercise	5.1 ± 1.8	3.6 ± 1.5	4.9 ± 1.5	0.286	0.99	0.485
24 h post-exercise	5.3 ± 1.4	4.7 ± 0.7	5.5 ± 0.7	0.704	0.99	0.322
48 h post-exercise	5.7 ± 2.4	3.8 ± 1.5	5.5 ± 1.9	0.233	0.99	0.321
72 h post-exercise	5.1 ± 1.2	4.6 ± 1.2	5.6 ± 0.8	0.99	0.99	0.410
TAS (mmol Trolox Equiv.·L^−1^)						
Pre-supplementation	1.3 ± 0.1	1.2 ± 0.3	1.3 ± 0.3	0.99	0.99	0.99
Pre-exercise	1.6 ± 0.2	1.2 ± 0.2	1.3 ± 0.2	0.899	0.087	0.627
72 h post-exercise	1.3 ± 0.3	1.2 ± 0.1	1.1 ± 0.3	0.99	0.843	0.99
MDA (µM)						
Pre-exercise	41.3 ± 13.5	48.4 ± 11.0	39.2 ± 15.2	0.99	0.99	0.701
72 h post-exercise	47.5 ± 19.7	45.1 ± 13.1	52.4 ± 22.8	0.99	0.99	0.99

AX: astaxanthin, CK: creatine kinase, LDH: lactate dehydrogenase, MDA: malondialdehyde, TAS: total antioxidant status; p^a^ = AX12 versus AX36, p^b^ = AX12 versus PLC, p^c^ = AX36 versus PLC, *: p < 0.05

## Discussion

In this study, we investigated whether four weeks of AX supplementation at different doses (12 mg·day^−1^ and 36 mg·day^−1^) would improve markers of antioxidant capacity (TAS, uric acid and MDA) and mitigate exercise-induced skeletal muscle damage (CK and LDH) in active males. The results indicate that both 12 mg and 36 mg daily doses of AX similarly reduced plasma CK activity following an acute bout of exhaustive resistance exercise, without significantly affecting antioxidant capacity markers or perceived muscle soreness. These findings suggest that 12 mg·day^−1^ of AX is sufficient to mitigate the acute muscle damage response to exercise, with no additional benefit observed at the higher dose.

Astaxanthin is a carotenoid with powerful antioxidants (Naguib, 2000) and anti-inflammatory properties (Andersen et al., 2007; Cao et al., 2023). An early study by Miki (1991) indicated the antioxidant activity of AX was 10 times greater than other carotenoids, such as zeaxanthin, lutein, canthaxanthin, and beta-carotene and had strong capabilities in quenching singlet oxygen and scavenging free radicals effectively (Miki, 1991). Therefore, recent research has raised questions as to whether AX could counteract exercise-induced muscle damage, which causes discomfort and impairs athletic performance, thereby potentially improving exercise capacity. However, the findings of these studies vary (Brown et al., 2017; Cao et al., 2023; Wong et al., 2020), with most of them reporting no impact of AX (6–20 mg·day^−1^) on exercise performance (Barker et al., 2023; Res et al., 2013), CK (Klinkenberg et al., 2013), or MDA (Klinkenberg et al., 2013) following resistance exercise in humans.

In the present study, although no significant change was evident in antioxidant capacity markers or LDH activity among our participants after four weeks of AX supplementation, we found lower CK activity after a single bout of 1-RM arm exercise in active males who had consumed AX (12 mg·day^−1^ or 36 mg·day^−1^) for four weeks. It is worth noting herein that although we did not measure plasma AX concentrations, previous research has shown supplementing with 2, 8, and 20 mg·day^−1^ of AX for four weeks significantly increased plasma AX concentrations in humans (Park et al., 2010; Res et al., 2013), suggesting a potential increase in plasma AX concentrations in our participants. Based on this, the observed reduction in CK activity in the present study was likely to be potent antioxidant activity associated with increased plasma AX concentrations (Naguib, 2000). Indeed, these increased AX concentrations may have contributed to the stability of the cell membrane, in addition to the scavenging of free radicals (Guerin et al., 2003). Similarly to our findings, Djordjevic et al. (2012) showed lower post-exercise CK activity after a 2-h bout of intense sport-specific exercise in elite youth soccer players (age: 17.9 ± 0.7 years) who had previously consumed AX (4 mg·day^−1^) for 90 days (Djordjevic et al., 2012). Barker et al. (2023) also demonstrated that four weeks of AX supplementation (12 mg·day^−1^) decreased subjective markers of DOMS without detriment to performance after the 1-RM test for the leg-press, followed by five sets of ten repetitions at 65% of the 1-RM, in resistance-trained men (age: 22.6 ± 2.2 years) (Barker et al., 2023). In addition, Choi et al. (2011) reported that twelve weeks of AX supplementation (20 mg·day^−1^) enhanced baseline total antioxidant capacity and reduced MDA concentrations in sedentary individuals with overweight (age: 30.6 ± 9.5 years) (Choi et al., 2011). Contrary to our non-significant change in MDA concentrations, the reduction in baseline MDA concentrations observed in the Choi and colleagues’ study (2011) may be attributable to the longer supplementation duration (twelve weeks vs. four weeks) and participants’ groups (normal weight individuals vs. those with overweight).

Conversely, several studies examining the impact of AX supplementation on markers of muscle damage, exercise performance, and muscle soreness after resistance- and endurance-based exercise have reported limited effects of AX supplementation on these variables in humans. More specifically, for instance, Bloomer and colleagues (2005) reported that three weeks of AX supplementation did not affect muscle soreness, CK activity, and muscle performance before and through 96 h post-eccentric exercise (10 × 10 repetitions at 85% of 1-RM) in resistance-trained men (age: 25.1 ± 1.6 years). Similarly, Waldman et al. (2023) recently demonstrated that four weeks of AX supplementation at a dose of 12 mg·day^−1^ had no significant effect on markers of muscle damage, inflammation, or DOMS following an exercise- induced muscle damage protocol consisting of drop jumps (20 repetitions × 5 sets) in resistance-trained males (age: 23.4 ± 2.1 years). Furthermore, four weeks of AX supplementation at 20 mg·day^−1^ was reported not to affect markers of antioxidant capacity, markers of inflammation or exercise-induced skeletal muscle damage after a single session endurance exercise involving 60 min of cycling, in well trained male cyclists (age: 25 ± 5 years) (Klinkenberg et al., 2013). A recent study by Nieman and colleagues (2023) also reported that four weeks of AX supplementation (8 mg·day^−1^), taken before running for 2.25 h at approximately 70% maximal oxygen uptake, did not affect exercise-induced muscle soreness or muscle damage in male and female runners (age: 42.2 ± 2.8 years) (Nieman et al., 2023). However, the supplementation did counteract exercise-induced reductions in 82 plasma proteins involved in immune functions, including defence responses, complement activation, and humoral immune system responses, during a 24-h recovery period (Nieman et al., 2023). This finding suggests that short-term AX supplementation (8 mg·day^−1^ for four weeks) could provide immune support for athletes. However, further research is needed to draw a solid conclusion on this potential impact of AX in humans. These reported inconsistencies in the ergogenic effects of AX might be attributed to dosage (2, 4, 8, 12 mg·day^−1^), duration of supplementation, types of exercise (endurance and resistance-based exercise), differences in the protocol for inducing exercise-induced muscle damage, and participants’ groups studied, causing the lack of consensus regarding the efficacy of AX supplementation in reducing exercise-induced muscle damage and antioxidant capacity markers.

The generalizability of our results is subject to certain limitations. A key limitation of our study is the absence of a pre-supplementation exercise test followed by blood sampling. Although we assessed blood markers related to muscle soreness after the post-supplementation exercise test, the absence of comparable pre-intervention data limited our ability to evaluate changes relative to baseline. Moreover, the sample size in this study was relatively small, which limits the statistical power and reduces the generalizability of the findings; therefore, future research with larger, more diverse samples is needed to confirm and extend these results. Additionally, the lack of a crossover design prevented within-subject comparisons under different conditions. These factors should be considered when interpreting the findings and highlight the need for further research with more robust experimental designs.

## Conclusions

Four weeks of AX supplementation at doses of 12 mg·day^−1^ and 36 mg·day^−1^ similarly attenuate plasma CK activity following a single session of resistance-based exercise in active males, while exerting minimal effects on markers of muscle damage, antioxidant capacity, and perceived muscle soreness. These findings suggest that a daily dose of 12 mg of AX may be sufficient to mitigate the acute muscle damage response to exercise, particularly in individuals engaged in repeated training cycles. For young, healthy individuals engaged in resistance exercise, incorporating a daily supplementation of 12 mg of AX could offer a strategy to alleviate exercise-induced muscle damage and reduce muscle soreness without adverse effects. This supplementation may prove beneficial not only for recreational athletes but also for those involved in competitive sports, where optimal recovery between training sessions and competitions is crucial. In particular, for athletes in sports requiring repeated high-intensity training, such as strength sports, endurance events, or mixed-modality training, AX supplementation might support faster recovery, reduce muscle pain, and help maintain peak performance levels during intense training phases or competition. However, more research is needed to better understand the underlying mechanisms through which AX supplementation exerts its effects on muscle damage, muscle performance, and recovery. Future studies should also explore the impact of AX supplementation over extended periods (e.g., > twelve weeks) and at varying dosages to determine optimal dosage ranges for maximal benefit. Additionally, comparative research involving different exercise modalities, such as resistance vs. endurance training, will be important for identifying the specific contexts in which AX supplementation is most effective in modulating markers of muscle damage, muscle soreness, and antioxidant status. Expanding research to include diverse populations (e.g., older adults, athletes, or individuals with pre-existing conditions) would also provide a more comprehensive understanding of the generalizability and potential therapeutic uses of AX supplementation in muscle recovery and overall exercise performance.
